# Supplementary material for: Impact of Thermally Inactivated Non-Saccharomyces Yeast Derivatives on White Wine
Source: Foods. 2024 Aug 22;13(16):2640. doi: 10.3390/foods13162640 (PMC11353631; doi:10.3390/foods13162640)
Supplement: Supplementary file 1 [file foods-13-02640-s001.zip › foods-3127663-supplementary.pdf]

Table S1. Phenolic composition of Trebbiano Toscano white wines added with TIYs.

	**t_R (min)**	**CT**	**SC**	**MP**	**SL**	**TD**
Gallic acid	6.03	44092^{a} ± 6196	47840^{ab} ± 700	44672^{ab} ± 963	45601^{ab} ± 2275	52548^{b} ± 1201
Unknown 1	6.46	189928^{b} ± 2539	186296^{b} ± 3904	182574^{b} ± 5620	135252^{a} ± 46246	112596^{a} ± 17005
Unknown 2	7.05	43237^{b} ± 1983	44132^{b} ± 48	42684^{ab} ± 286	41200^{ab} ± 3497	37942^{a} ± 3081
p-cumaric acid	8.81	203149^{b} ± 8343	192012^{b} ± 2220	193435^{b} ± 1509	175617^{b} ± 13113	122254^{a} ± 25040
Unknown 3	11.20	325320^{b} ± 19570	317004^{b} ± 5097	259079^{ab} ± 4779	264957^{ab} ± 9079	214821^{a} ± 64463
Unknown 4	15.91	101139^{b} ± 1228	82005^{ab} ± 1095	73700^{ab} ± 2536	65532^{a} ± 21453	91912^{ab} ± 17599
Unknown 5	16.50	101032^{b} ± 1072	94994^{ab} ± 1596	94978^{ab} ± 1710	87640^{a} ± 3507	92766^{a} ± 7413
trans-caftaric acid	18.56	906537^{b} ± 9169	906943^{ab} ± 8626	874349^{b} ± 45850	888951^{ab} ± 14272	870992^{a} ± 6554
procyanidin B1	20.10	155798^{ab} ± 12739	160419^{b} ± 6942	135635^{ab} ± 25472	145517^{ab} ± 19427	130474^{a} ± 5310
(+)-catechin	21.50	112279^{a} ± 8261	113298^{a} ± 17863	94713^{a} ± 5054	120447^{a} ± 9302	106397^{a} ± 5036
(-)-epicatechin	24.48	67914^{a} ± 1778	70774^{a} ± 4027	68407^{a} ± 1160	68238^{a} ± 9785	69839^{a} ± 9989
caffeic acid	25.10	68873^{ab} ± 4267	75631^{b} ± 7645	67580^{ab} ± 4873	68661^{ab} ± 9810	63553^{a} ± 8689
cutaric acid	26.00	72978^{a} ± 670	80928^{a} ± 15401	71953^{a} ± 1781	68501^{a} ± 894	66760^{a} ± 4221

Values are expressed as peak area of the chromatogram recorded by RP-HPLC DAD at 280 nm. Data are average ± standard deviation of three independent replicates. Different letters in the same row indicate values significantly different. LSD, Least Significant Difference Test; 95% Significance Level.
